# “UFO procedure” for massive aortic and mitral annular calcification involving left atrial and ventricular myocardium: a potential radical solution

**DOI:** 10.1186/s13019-023-02267-5

**Published:** 2023-05-25

**Authors:** Leonhard Wert, Miralem Pasic, Roland Heck, Karel M. Van Praet, Jörg Kempfert, Stephan Jacobs, Volkmar Falk

**Affiliations:** 1 Department of Cardiothoracic and Vascular Surgery, Deutsches Herzzentrum der Charité (DHZC), Augustenburger Platz 1, 13353 Berlin, Germany; 2 DZHK (German Center for Cardiovascular Research), partner site Berlin, Berlin, Germany; 3 Department of Cardiothoracic Surgery, Charité − Universitätsmedizin Berlin, corporate member of Freie Universität Berlin, Humboldt-Universität zu Berlin, and Berlin Institute of Health, Berlin, Germany; 4 Department of Health Sciences and Technology, ETH Zurich, Zurich, Switzerland

## Abstract

**Background:**

The “UFO procedure” was initially developed as a surgical option to enlarge the aortic annulus in patients requiring valve replacement. This technique can be employed to treat extensive endocarditis located in the intervalvular fibrous body (IVFB). One of the indications for a "UFO procedure" is massive aortic and mitral valve calcification. It is a challenging surgical procedure with a high risk of intraoperative complications.

**Case summary:**

We present a 76-year-old male patient with massive aortic and mitral valve calcification involving the left atrium, the left ventricle and the left ventricular outflow tract. Both valves exhibited severe stenosis and moderate to severe regurgitation. The left ventricle was hypertrophic and the left ventricular ejection fraction was > 55%. The patient was prediagnosed with persistent atrial fibrillation. The risk of death following heart surgery (EuroSCORE II) was calculated as 9.21%. We successfully performed a so-called “UFO procedure” including replacement of both valves without annular decalcification to avoid atrioventricular dehiscence. We enlarged the IVFB and replaced the non-coronary sinus of Valsalva with doubled bovine pericardium. The left ventricular outflow tract was decalcified. The patient was transferred to a local hospital on the 13th postoperative day.

**Conclusion:**

Successful surgical treatment to this extent was demonstrated for the first time. Due to the high perioperative mortality, the surgical treatment of patients with this constellation would be refused in most cases. In our patient, the preoperative imaging showed extreme calcification of both valves and the surrounding myocardium. Excellent preoperative planning and a highly experienced surgical team is necessary.

**Supplementary Information:**

The online version contains supplementary material available at 10.1186/s13019-023-02267-5.

## Introduction

Most of us are familiar with the term “UFO” meaning an “unidentified flying object”. When used in connection with a surgical technique, it is meant to emphasize the complexity of this double valve replacement procedure. The term “UFO procedure” was coined by a resident who observed a surgery at the Toronto General Hospital. Other terms, including “Commando operation” or “Combat procedure”, have also been used to describe this surgical technique [1].

The "UFO procedure" was first mentioned by Manouguian in 1976. It was initially designed to enable enlargement of a very small aortic annulus [2]. David TE et al. rediscovered this technique in Toronto. They published outcomes of a larger cohort of patients [3]. Today, this surgical technique has proven successful in patients with double valve endocarditis with or without an abscess in the intervalvular fibrous body [4]. Another indication is massive calcification of the aortic and the mitral annulus with destruction of the surrounding tissue [5]. Other centres prefer using this procedure for complex reoperations. In spite of these advances the overall postoperative prognosis remains poor [6, 7].

## Case presentation

### Patient details

In May 2020, a 76-year-old male patient (170 cm, 70 kg, BMI 24.2 kg/m^2^, BSA 1.81 m^2^) presented with dyspnoea consistent with NYHA class III. The patient’s history included persistent atrial fibrillation under oral anticoagulation with phenprocoumon, as well as arterial hypertension and hyperlipidaemia. Coronary artery disease was excluded. The patient was being treated with an ACE inhibitor, rosuvastatin, and rivastigmine due to mild Alzheimer’s disease. Bronchial asthma was still to be clarified and was left untreated for the time being. Preoperative blood tests were normal except for a slightly elevated white cell count of 11.1 × 1000/µL with neutrophilia.

**Fig. 1:**
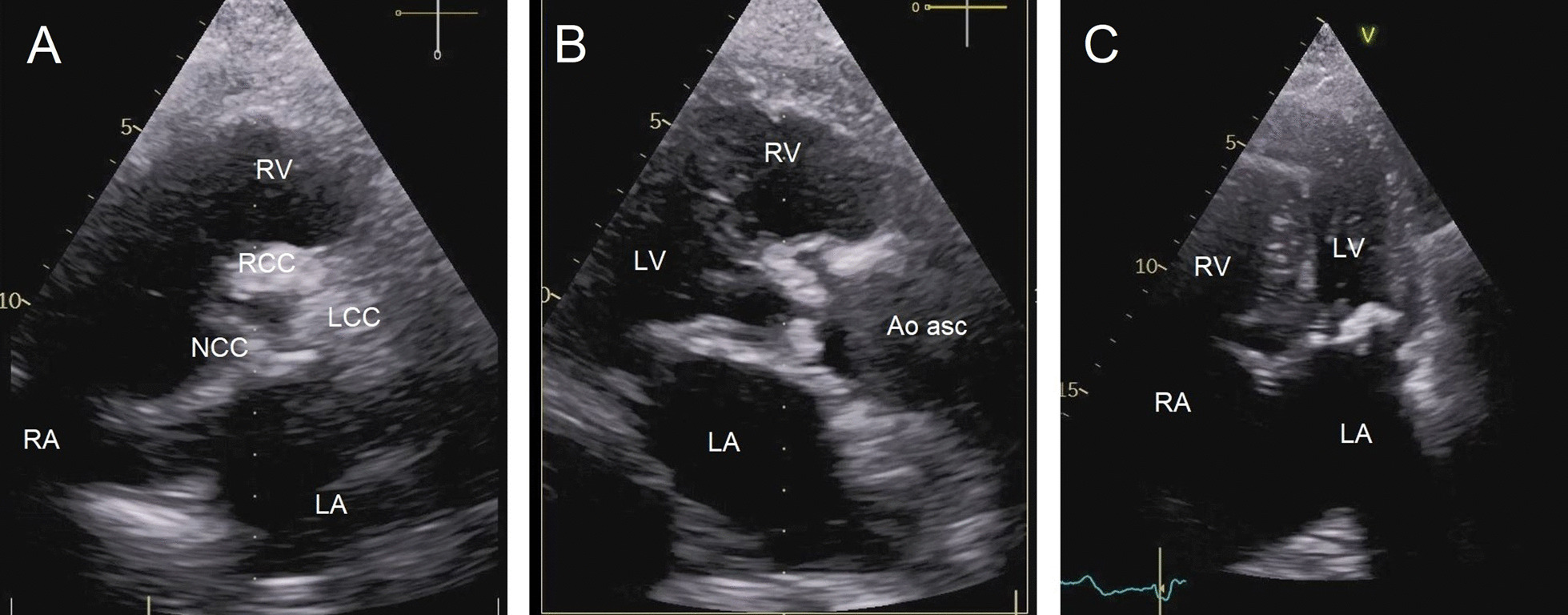
Preoperative transthoracic echocardiogram images. Parasternal short-axis view (**A**) and parasternal long-axis view (**B**) of the calcified aortic valve. Apical four-chamber view showing left ventricular hypertrophy and severe calcification of the mitral valve (**C**). Ao asc (ascending aorta), LA (left atrium), LCC (left coronary cusp), LV (left ventricle), NCC (non-coronary cusp), RA (right atrium), RCC (right coronary cusp), RV (right ventricle)

Transthoracic echocardiogram revealed left ventricular hypertrophy with moderate dilatation with a left ventricular end-diastolic diameter (LVEDD) of 3.9 cm and a left ventricular posterior wall thickness (LVPWd) of 0.91 cm. The interventricular septal thickness at diastole (IVSD) was 1.4 cm. The left ventricular systolic function was preserved (LVEF of 0.55 using Simpson’s biplane method of discs). The aortic root dimensions were normal. The right ventricle was normal in size and function by visual estimation. The left atrium was significantly dilated. The right atrial size was normal. The aortic annulus measured 26 mm. The tricuspid aortic valve exhibited severe stenosis with a median pressure gradient of 29 mmHg and moderate regurgitation with a pressure half-time of 120 ms (Fig. 1; Additional files 1, 2, 3: Video 1–3).

**Fig. 2:**
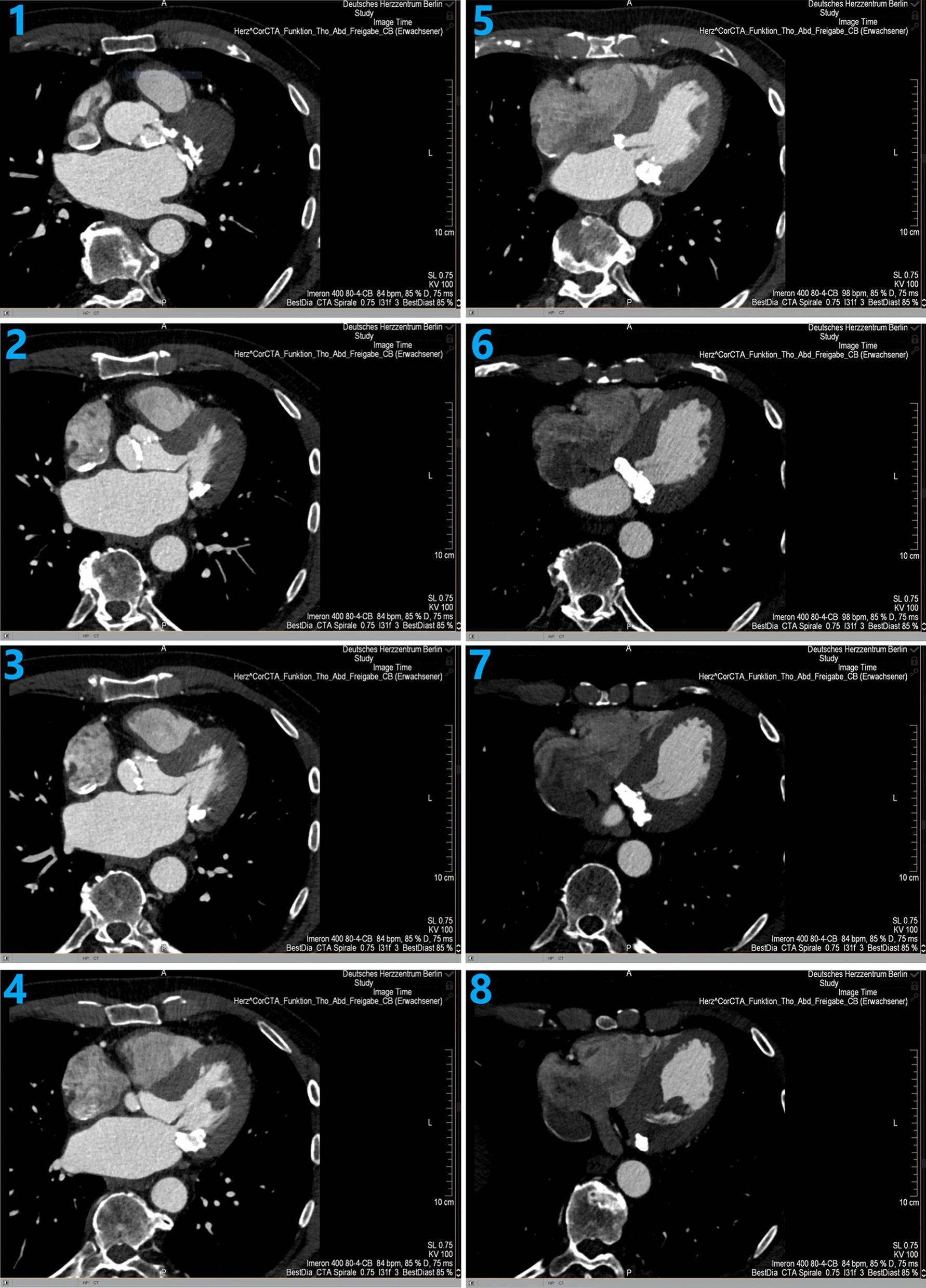
Preoperative CT scan showing massive calcification of the mitral valve and the mitral annulus with involvement of the left atrium, the left ventricle and the left outflow tract

The CT scan (Fig. 2, Additional file 4: Video 4) revealed severe mitral valve calcification (MAC) and in particular severe circular calcification of the native mitral valve annulus. The calcification propagated in the neighbouring myocardium both of the left ventricle and left atrium.

**Fig. 3:**
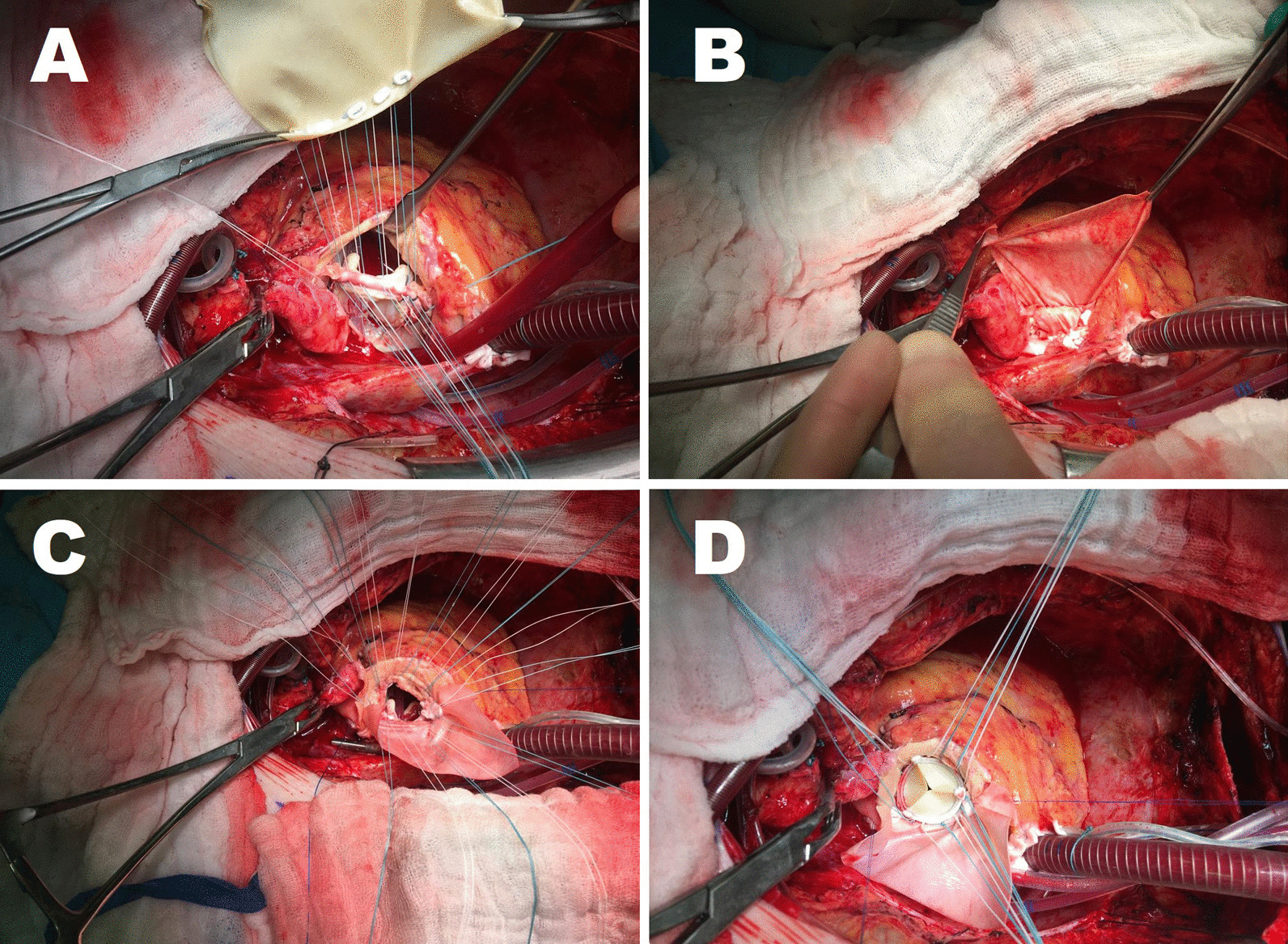
General technique of a “UFO procedure”. Intraoperative view on prepared sutures from doubled patch through mitral valve prosthesis (**A**). Intraoperative view on reconstructed left atrial roof using the double patch technique (**B**). Intraoperative view on prepared sutures for the aortic valve prosthesis (**C**). Intraoperative view on implanted aortic valve prosthesis (**D**)

During the "UFO procedure" the left atrial roof was opened and the incision was extended to the middle of the non-coronary part of the aortic annulus and further through the anterior mitral leaflet (AML). The calcified AML was excised and interfibrous trigones were partially decalcified. However, because the quality of the tissue was insufficient, the fixation of the doubled pericardial patch was not performed in the conventional way. Instead, a doubled patch was tailored to be larger than a standard one. This enables additional fixation of the patch in both adjacent parts of the former P1 and P3 region with the stitches put through the ring of the new mitral valve prosthesis. Importantly, while placing the stitches for the aortic valve replacement, the U-sutures were placed from outside the aorta through the aortic wall and additionally through the pericardial patch in order to prevent any possible gaps and consecutive bleeding in this region (Fig. 3).

One of our modifications of the "UFO procedure" is to avoid incision of the right atrium. Leaving the right atrium intact significantly lowers the risk of rhythm complications involving the sinus node and the AV node. The exposition of the situs can be improved when you use a two-stage venous cannulation of the right atrium. The two-stage cannula can be pulled to open the left atrial roof more. Furthermore, in our opinion, in some instances venous drainage is better than after bicaval cannulation [4, 5]

Both intrafibrous trigones were also calcified. Both leaflets were calcified and showed restricted leaflet motion. The medio-lateral size of the annulus was 34 mm. There was severe stenosis with moderate regurgitation and a centrally directed jet of the mitral valve. The median pressure gradient of the mitral valve was 7.1 mmHg. The mitral valve area measured by planimetry was 1.6 cm^2^ (Fig. 1; Additional file 3: Video 3). There was mild tricuspid regurgitation.

### Surgical procedure

After induction of anaesthesia, a median sternotomy was performed. We placed the arterial line in the aortic arch using the Seldinger technique and a two-stage venous cannula in the right atrium. Cardiopulmonary bypass was established. The ascending aorta was cross-clamped and cardioplegia was administered directly into the coronary ostia. After performing an oblique aortotomy, we resected the severely degenerated and calcified cusps of the aortic valve. The calcification extended from the ascending aorta to the left ventricular outflow tract (LVOT). The in-situ measurement revealed a significantly smaller aortic annulus. We decalcified the aortic annulus. We removed the calcification in the LVOT in the way that the anterior mitral leaflet (AML) was excised. Additionally limited decalcification was performed in the region of the free muscular wall of the LVOT to enable safe and precise placement of the stitches for the aortic valve implantation. A separate incision was made between the non-coronary and the left coronary sinuses of Valsalva to the left atrial roof and the AML. The AML was vertically exposed to the surgeon. As expected, the mitral annulus exhibited annular calcification. Both leaflets of the mitral valve were calcified. The myocardium of the posterior wall of the mitral valve, the left atrium and the left ventricle exhibited severe calcification. We resected both leaflets. The extensive calcification of the mitral valve propagated from the annulus of the mitral valve into the neighbouring myocardium of the left atrium and the left ventricle. The calcifications of the native mitral valve annulus and of the neighbouring myocardium were not removed; they were left completely untouched. An only limited removal of the calcification was undertaken in the region of the posterior leaflet in order not to disturb the placement of the new mitral valve prosthesis. To this end, only as much of the protruding calcifications as necessary were removed.

The conventional pledgeted mitral valve sutures were not used for the mitral valve implantation. Instead, we applied polypropylene 2–0 U-sutures supported with small pieces of bovine pericardium (St. Jude Medical Inc., USA). The bovine pericardial pledgets were cut from the pericardial patch to the smaller pieces (10 × 15 mm) on the operating table immediately before surgery.

The polypropylene U-sutures were placed from the left ventricular cavity through the calcified native mitral annulus towards the left atrium. In order to prevent possible bleeding through the suture holes into the left ventricular myocardium (and thereby prevent possible atrioventricular dehiscence), additional strips of bovine pericardium (width of the strips about 8–10 mm) were used. The strips were applied as additional support for the U-sutures on both the ventricular and the atrial side. To achieve this, the needle of the polypropylene U-sutures (pledgeted with 10 × 15 mm pieces of pericardial patches) were first placed through the pericardial strip on the ventricular side, then through the calcified native mitral annulus into the left atrium, and finally and additionally through the second pericardial strip on the atrial side of the calcified mitral annulus. This technique stabilises the fragile tissue and prevents atrio-ventricular dehiscence.

In the next step we reconstructed and closed the left atrial roof with one half of a doubled patch of bovine pericardium. The other half of the doubled patch was turned over like a butterfly to reconstruct the intervalvular fibrous body (IVFB) and to replace the non-coronary sinus of Valsalva. The second half of the doubled patch also served to close the aortotomy. This double patch technique allowed for enlargement of the aortic and the mitral annulus. We then used biological prostheses to replace the aortic (25 mm diameter, Trifecta, St. Jude Medical Inc., USA) and the mitral valve (33 mm diameter, BioMitral, BioIntegral Surgical Inc., Canada). After thorough surgical haemostasis, the patient was weaned from cardiopulmonary bypass without inotropes. The thorax was closed using the standard technique. The clamping time was 142 min, the perfusion time 167 min.

The patient was extubated on the first postoperative day and was transferred to the rehabilitation hospital on the 13th postoperative day. A postoperative control echocardiography confirmed a correct position and normal valve function with no paravalvular leak from either of the prostheses (Additional files 5, 6, 7, 8 and 9: Video S1–S5). Left ventricular size (LVEDD 4.4 cm) and left ventricular ejection fraction was maintained at 0.55. There was no tricuspid regurgitation.

## Discussion

There are two “Manouguian techniques”: one is used to enlarge the aortic valve annulus in preparation for aortic valve implantation (this is usually referred to as "the Manouguian technique"), while the second is used for double (aortic and mitral) valve replacement. The latter technique was called “UFO procedure” or “commander procedure”, or also “Commando procedure”, but –wrongly– not the “Manouguian technique”. Both Manouguian methods are very old techniques that were developed as a means of enlarging either the aortic annulus alone or both the aortic and mitral annuli. Manouguian himself first tried out this technique in a canine model in the 1970s [2]. After surgical failures with torn patches he developed a patch technique in 1980 [8]. In the late 1990s David et al. popularised this procedure for patients with endocarditis for radical debridement of the intervalvular fibrous body with an abscess. The Toronto colleagues called this technique “UFO procedure” [3]. The “Manouguian technique” describes the basic surgical method using the patch technique with the indication for enlargement of the aortic and/or mitral annulus.

This technique can be used to treat extensive endocarditis with or without an abscess located in the IVFB. Previous studies have described an operative mortality of up to 24% in patients with a paravalvular abscess [3, 5, 6, 9]. Most of the published studies focus on the surgical treatment of patients with endocarditis in a last-resort setting.

Data concerning patients with massive calcification of the IVFB are scarce. This is likely due to the fact that the absolute number of patients with massive calcification is generally low. It is possible that there is a great hesitation to perform this complex procedure. De Oliveira et al. evaluated 76 patients undergoing a UFO procedure. In 24 of the 76 patients, the indication for the surgery was extensive calcification of the mitral annulus and the IVFB. Three patients were reoperated due to prosthetic valve endocarditis. The long-term survival of all 76 patients after 5 and 10 years was 71 ± 6% and 50 ± 9% [6]. In a small cohort of 14 patients with massive calcification, Forteza A et al. reported a hospital mortality rate of 7.1%. The survival rate after 1, 5 and 10 years was 92.3%, 84.6% and 78.6%, respectively [5].

All studies performed to date are limited because they are retrospective and involve a small number of patients. Furthermore, several modifications of the "UFO procedure" exist; therefore, comparability is not fully guaranteed. Small surgical details are center-dependent, i.e. bicaval cannulation versus two-stage cannulation, incision of both atria versus only left atrium. In this particular case, it was the the surgeon's decision to perform patch reconstruction with minimal LVOT debridement instead of a full debridement [10].

The main point of this specific case is that we used the “UFO procedure” for an unusual indication, namely for MAC, severe calcification of the mitral valve with circular calcification of the annulus propagating into the neighbouring myocardium of the left ventricle and the left atrium in a patient with double valve pathology (aortic and mitral). The “UFO procedure” enabled us to implant larger valves than those that could be implanted under these circumstances and, additionally, made the actual procedure easier than the conventional one in this patient. At the same time the “UFO procedure” reduced the risk of the procedure as whole. We would like to stress that what made this particular case so unique was the way of dealing with the calcified mitral annulus. We did not use conventional pledgeted mitral valve sutures. Instead, we used polypropylene U-sutures supported with small bovine pericardial pieces that might be better suited in this particular situation with extreme calcifications.

In regard to our surgical technique, we performed the “UFO procedure” with our “Berlin modification”. It is well known that several modifications of the “UFO procedure” exist. Small surgical details are centre-dependent, i.e. bicaval cannulation versus single two-stage cannulation or incision of both atria versus only the left atrium. In general, one of our principles is to avoid incision of the right atrium. This might reduce the risk of postoperative rhythm complications involving the sinus node and the AV node. Furthermore, we routinely use only a single venous cannula for venous drainage; we put a two-stage cannula through the right appendage of the right atrium. This enables a better view of the operating field and better exposure. Furthermore, our experience shows that venous drainage with a single cannula usually tends to be better than with a separate bicaval cannulation.

Conduction disturbances are a common complication after a "UFO procedure". In a case series of four patients who underwent "UFO procedure", Chen et al. described a postoperative pacemaker obligation for all patients [9]. One of our modifications of the UFO procedure is to absolutely avoid incision of the right atrium. While an opened right atrium provides better exposure, it also significantly increases the risk for rhythm complications involving the sinus node and the AV node. Two-stage venous cannulation of the right atrium can improve the exposure. The two-stage cannula can be pulled to open the left atrial roof more. Furthermore, we believe that, in some instances, venous drainage is better than after bicaval cannulation [7, 10]. In addition, placing the stitches from outside the aorta to inside the region of the commissure between the non-coronary and the right coronary sinus of Valsalva—as described in this case—might be helpful in preventing rhythm disturbances.

In our case this procedure is a radical surgical solution. Despite the overall high-risk situation, the "UFO procedure" remains the only option for patients with heavily calcified aortic and mitral annuli. It is an exceedingly challenging technique with a high risk of intraoperative complications. It must be stressed that this complex case should be handled on by an experienced surgeon. Therefore, the classical double valve replacement is a possible alternative and the “UFO procedure” is not imperative in this situation. However, we believe that for surgeons who are familiar with the “UFO procedure”, it is a simpler choice and less dangerouse than the conventional double valve replacement. Since the MAC was not removed in the described procedure, the surgical risk was significantly reduced even though a large mitral valve was implanted. This would not be possible without decalcification if a conventional double valve replacement were performed.

## Supplementary Information

**Additional file 1.** Video 1 Preoperative transthoracic echocardiogram images. Parasternal short-axis view (right side) and parasternal long-axis view (left side) showing movement and severe calcification of the aortic valve.

**Additional file 2.** Video 2 Preoperative colour Doppler parasternal long-axis view showing severe aortic stenosis.

**Additional file 3.** Video 3 Preoperative colour Doppler apical four-chamber view showing severe calcification and stenosis of the mitral valve.

**Additional file 4.** Video 4 Preoperative computed tomography angiography.

**Additional file 5.** Video S1 Postoperative parasternal long-axis view showing mitral valve replacement without colour Doppler imaging.

**Additional file 6.** Video S2 Postoperative colour Doppler parasternal long-axis view showing mitral valve replacement with no transvalvular or paravalvular regurgitation.

**Additional file 7.** Video S3 Postoperative continuous wave Doppler in the parasternal long-axis view.

**Additional file 8.** Video S4 Postoperative apical five-chamber view showing aortic valve replacement without colour Doppler imaging.

**Additional file 9.** Video S5 Postoperative continuous wave Doppler in the apical five-chamber view.

## Data Availability

All data is available in electronic medical record.
